# Splitpea: a Python package for protein-protein interaction network rewiring analysis due to alternative splicing

**DOI:** 10.1093/bioinformatics/btag154

**Published:** 2026-04-08

**Authors:** Jeffrey Zhong, Alyssa Cantu, Ruth Dannenfelser, Vicky Yao

**Affiliations:** Department of Computer Science, Rice University, Houston, TX 77005, United States; Department of Computer Science, Rice University, Houston, TX 77005, United States; Department of Computer Science, Rice University, Houston, TX 77005, United States; Department of Computer Science, Rice University, Houston, TX 77005, United States; Ken Kennedy Institute, Rice University, Houston, TX 77005, United States; Rice Synthetic Biology Institute, Rice University, Houston, TX 77005, United States

## Abstract

**Summary:**

Splitpea takes skipped exon event data at the sample or differential expression level from SUPPA2 and rMATS and maps potential changes to protein-protein interaction (PPI) network rewiring events. It handles a variety of input formats via an easy-to-install Python package, including percent spliced in values comparing two conditions, skipped exon counts, or precalculated exon usage statistics between experimental conditions. In each case, Splitpea produces rewired network graphs, edge and gene-level summary statistics, and Cytoscape- or Gephi-ready files for easy visualization, allowing users to find PPIs potentially disrupted or increased by alternative splicing.

**Availability and implementation:**

Source code and accompanying documentation can be found on Github (https://github.com/ylaboratory/splitpea-package), released under a BSD 3-clause license for open-source use, and the Splitpea package is installable via PyPI.

## 1 Introduction

Alternative splicing generates diverse protein isoforms that reshape protein-protein (PPI) interactions, fundamentally altering cellular functions in health and disease (Scotti and Swanson 2016, Baralle and Giudice 2017). While RNA sequencing can capture splicing patterns across varied conditions, isoform-isoform specific PPI networks are still restricted in their resolution (Yang *et al.* 2016), necessitating alternative approaches to link splicing changes with PPIs. The Splitpea method (Dannenfelser and Yao 2024) was introduced to detect potential rewiring events in PPI networks based on splicing patterns in individual samples. The key intuition underlying Splitpea is that splicing changes can disrupt or reconstitute important protein domains that mediate PPIs and thus suggest whether a PPI is likely lost or gained. Although the per-sample view provided by the original Splitpea method is valuable, many splicing studies focus on differential exon usage between conditions (e.g., case versus control). Commonly used tools to enumerate splicing events and quantify changes between conditions, such as SUPPA2 (Trincado *et al.* 2018) and rMATS (Wang *et al.* 2024), do not explicitly translate per-event PSI (percent spliced in) values back into potential changes at the protein level.

Despite the availability of tools for analyzing alternative splicing and its impact on protein function and interactions, existing applications differ substantially in how they connect splicing changes to PPI networks. Most approaches emphasize isoform-level characterization or transcript abundance, rather than directly modeling interaction-level rewiring driven by differential exon usage. IsoformSwitchAnalyzeR (Vitting-Seerup and Sandelin 2019), for example, is largely transcript-centric, focusing on detecting isoform switches and annotating predicted functional consequences, such as coding potential and domain changes, rather than explicit changes in PPIs. PPIXpress (Will and Helms 2016) and PPICompare (Do *et al.* 2025) build condition-specific PPIs by integrating transcript expression to assess which interactions are likely present, but they are not designed to take common differential exon usage outputs from tools such as SUPPA2 or rMATS as primary inputs. DIGGER (Louadi *et al.* 2021) provides an interface for exploring how isoform variation may relate to protein interactions, but it is mainly geared toward interactive exploration and visualization, rather than systematic analysis of splicing-driven PPI changes across conditions or relative to a background control.

To address interaction-level rewiring directly, we previously introduced Splitpea (Dannenfelser and Yao 2024) as a per-sample framework for linking splicing changes to PPIs. Here, we extend the original method into a fully packaged and easily installable implementation that supports multi-sample and multi-condition analyses, directly ingests rMATS and SUPPA2 outputs, bundles PPI and DDI reference datasets, and provides built-in visualization and standardized exports. All features are bundled as a pip-installable package on PyPI (pip install splitpea) and are accessible via a command-line interface or as a Python library. The Splitpea package includes protein-protein, domain-domain reference files, as well as normal human tissue splicing control data, to build rewired network maps. In addition to built-in network visualization and analysis functions, the package also provides exportable Cytoscape (Shannon *et al.* 2003) and Gephi (Bastian *et al.* 2009) files to explore resulting networks. In general, the Splitpea package enables users to move seamlessly from standard differential exon statistics to finding potential interaction gains and losses at the individual-sample and multi-sample condition levels.

## 2 Materials and methods

Splitpea is a Python package released under the BSD 3-clause license. Source code and documentation are hosted on GitHub (https://github.com/ylaboratory/splitpea-package), and the package is installable via PyPI.

**Figure 1:**
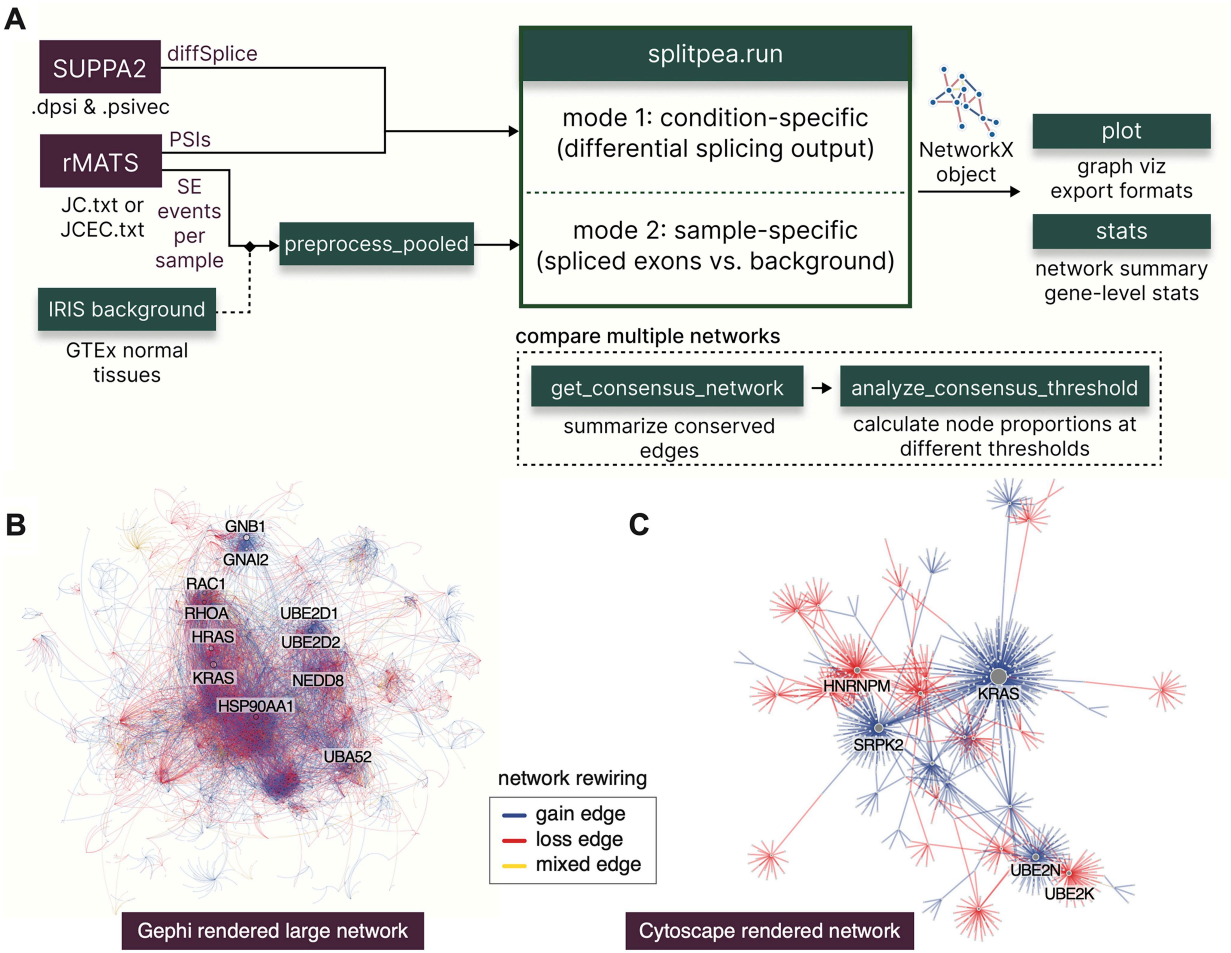
Overview and sample networks generated by the Splitpea package. (A) This overview diagram shows the process of starting from either SUPPA2 or rMATS input and using the Splitpea package to obtain desired outputs. Each element of the Splitpea package is highlighted in green. (B) The rewired network generated for the rMATS example, exported and rendered with Gephi, with node size proportional to degree. (C) The smaller rewired network generated from the SUPPA2 case study, exported and rendered with Cytoscape.

Splitpea, at its core, takes exon-skipping event outputs from SUPPA2 or rMATS and maps the effects of changes between different conditions onto a PPI network by considering which protein domains may be affected (Fig. 1A). Because exon-domain-protein relationships are many-to-many, exon-skipping events are first mapped to Pfam domains via genomic overlap; when multiple exons map to the same domain, the minimum exon-level ΔPSI is assigned to that domain, reflecting the assumption that partial domain loss may impair interaction capacity. The aggregated reference PPI network is overlaid with known domain-domain interactions (DDIs), such that a PPI is considered domain-mediated if at least one reported DDI exists between a pair of domains present in the two corresponding proteins. For each PPI supported by at least one affected DDI, domain-level ΔPSI values are used to characterize the interaction as a gain (consistent positive changes), loss (consistent negative changes), or chaos (mixed directions). Edge weights are computed as the mean of the contributing domain-level ΔPSI values, and edges are reported whenever at least one interacting protein contains a significant exon-skipping event; only DDIs mediating the given PPI contribute to edge classification and weighting. The final output is a rewired, weighted PPI network (see Algorithm 1, available as supplementary data at *Bioinformatics* online, for more details).

The package provides two primary modes of operation, sample-specific and condition-specific. Each mode relies on different internal calculations for splicing changes but produces the same outputs: an edge list file and a pickle file representing the NetworkX (Hagberg *et al.* 2008) graph. More specifically, in the sample-specific mode, rMATS-processed individual samples can be compared with a multi-sample reference background set following the algorithm outlined in Dannenfelser and Yao (2024). We provide an option to directly use GTEx normal tissue data (GTEx Consortium 2013) from the IRIS database (v2.0.0) (Pan *et al.* 2023) for sample comparisons. Background PSI values obtained via IRIS/GTEx are not tissue-matched by default; however, Splitpea allows users to supply tissue-matched or custom reference distributions.

For users interested in comparing against other background distributions, we also provide an option for using custom sample sets as reference backgrounds. While the package allows for any size sample background, we recommend that users provide at least 30 samples to ensure a more robust estimate of splicing changes relative to the sample of interest. In the condition-specific mode, Splitpea compares two defined groups of samples using the differential exon usage results directly from SUPPA2 or rMATS. rMATS junction reads (JC) or junction and exon reads (JCEC) output files can be used as input for both the sample-specific and condition-specific modes. Alternatively, the condition-specific mode can also use as input the .*psivec* and .*dpsi* output files from SUPPA2’s diffSplice function. Splitpea uses default differential splicing filters consistent with rMATS/SUPPA unless otherwise specified by the user. If not using rMATS/SUPPA defaults, the default parameters include a ΔPSI threshold of 0.05, an FDR cutoff of 0.05, and a minimum background sample size of ≥30. By default, Splitpea removes self-loops during plotting, as self-loops are uncommon across networks and their removal leaves degree distributions largely unaffected; however, users can optionally retain self-loops if desired (Figs 1 and 2, available as supplementary data at *Bioinformatics* online). Splitpea’s runtime scales linearly ($R^{2}$ = 0.99) with the number of differentially spliced exons (Table 1, available as supplementary data at *Bioinformatics* online).

By default, Splitpea includes pre-built reference files for human and mouse protein-protein and domain-domain interactions (defaulting to human). The human protein-protein interactions are aggregated from BioGRID (v4.4.207) (Chatr-Aryamontri *et al.* 2017), DIP (2017–02-05) (Salwinski *et al.* 2004), HIPPIE (v2.2) (Alanis-Lobato *et al.* 2017), HPRD (Release 9) (Keshava Prasad *et al.* 2009), HI-II (Rolland *et al.* 2014), IntAct (2022–04-18) (Orchard *et al.* 2014), iRefIndex (v18.0) (Razick *et al.* 2008), and MIPS (Nov 2014) (Pagel *et al.* 2005). Domain-domain interactions are compiled from 3did (v2017_06) (Mosca *et al.* 2014), DOMINE (v2.0) (Yellaboina *et al.* 2011), IDDI (2011-05-16) (Kim *et al.* 2012), and iPfam (v1.0) (Finn *et al.* 2014). However, users may provide custom reference files to override these defaults. Unless explicitly specified in the function parameters, Splitpea will automatically load the bundled reference PPI network, DDI catalog, Pfam coordinate definitions, and gene mapping and identifier table for the specified organism. Please refer to the package documentation for a complete list of arguments and supported file formats. To enhance the functionality of Splitpea, we also provide network analysis functions for common downstream tasks. For small networks, a native plotting function takes the pickled rewired network output and generates a spring layout plot in Python, with colored edges and node labels, using matplotlib. For larger networks (>50 nodes) and more graph visualization customization, we provide functionality for exporting Splitpea networks in Gephi (Bastian *et al.* 2009) and Cytoscape (Shannon *et al.* 2003) compatible formats. Additionally, we provide a statistics function that summarizes key information from the rewired network, including the number of various edge types and the following gene-level statistics: degree, normalized degree, and edge composition. The package can also compare and contrast multiple rewired networks through the consensus functions, which populate counts for edges conserved across the networks.

## 3 Example cases

To demonstrate the functionality of Splitpea, we applied the package to two distinct datasets using the condition-specific mode, which is likely the most common workflow for users starting with rMATS or SUPPA2 files. Tutorials for using the sample-specific mode can be found on GitHub in the package documentation.

### 3.1 rMATS input

First, we analyzed differential alternative splicing events identified by rMATS between two prostate cancer cell lines, metastatic versus non-metastatic, using previously published rMATS outputs (BioProject: PRJNA438990) (Lu *et al.* 2015, Wang *et al.* 2024). Considering both junction and exon counts, rMATS detects 308,739 skipped‐exon (SE) events, of which 17,835 pass a significance threshold of FDR <0.05, spanning 5,746 unique genes. We then ran Splitpea by specifying as input only the rMATS output file and the desired output path, leaving all other parameters (including the built in reference files and filters) at their defaults. This produced a large rewired network of 7,883 gain, 7,308 loss, and 1,821 mixed edges across 6,173 genes (Fig. 1B). We compared this Splitpea rewired network against IntAct’s (Orchard *et al.* 2014) experimentally supported isoform-specific PPIs curated for cancer. Despite the limited size of curated interactions (*n* = 730), we observed a significant overlap with the Splitpea network (hypergeometric *P* = .002; Table 2, available as supplementary data at *Bioinformatics* online), supporting the biological relevance of the inferred rewiring events.

In addition to identifying many more edges than previously curated, Splitpea also uncovers potential rewiring events involving 3,793 genes that rMATS did not flag as significantly spliced. These genes can highlight important functional changes caused by splicing, but themselves are not significantly differentially spliced. Specifically, among those genes, 212 were extensively rewired (degree ≥11), including several prostate cancer-associated genes, such as ERBB3 (0 gain, 92 loss, 8 mixed edges) (Gil *et al.* 2021, Vellky *et al.* 2024) and CHEK2 (43 gain, 0 loss, 2 mixed edges) (Dong *et al.* 2003, Seppälä *et al.* 2003). To illustrate a further potential downstream analysis we also ran rank-based gene set enrichment analyses using the GSEApy package (Fang *et al.* 2023) with Gene Ontology Biological Process (Ashburner *et al.* 2000) terms. We ranked the genes in two ways: by the number of gain-of-function and loss-of-function edges (breaking ties with normalized degree, then raw degree) and observed 149 and 112 significant functional changes, respectively (q<0.05 and NES>0; Table 3, available as supplementary data at *Bioinformatics* online). Specifically for gain edges, the top enriched pathways included mRNA stabilization, negative regulation of mRNA catabolic process, and U2-type prespliceosome assembly, consistent with prior studies showing that alternative splicing and dysregulation of RNA/spliceosome machinery are hallmarks of prostate cancer progression and therapeutic resistance, making RNA-processing programs biologically relevant readouts in this setting (Rawat and Heemers 2024). For loss edges, the top enriched terms included Fc-gamma receptor signaling, epidermal growth factor receptor signaling, and Rho protein signal transduction, highlighting disruption of core signaling pathways that have all been previously implicated in previous studies on prostate cancer progression and metastasis (He *et al.* 2022, Das *et al.* 2025). To visualize the network, we used Splitpea’s built-in plotting function to export the network as a TSV file compatible with Gephi. With Gephi we generated the final publication quality figure by clustering using the OpenOrd layout, scaling nodes based on degree, coloring edges by type using the suggested edge color annotations included in the TSV, and adjusting font and opacity settings (Fig. 1B).

### 3.2 SUPPA2 input

Next, we applied our package to SUPPA2-derived splicing events comparing early versus late erythroblast differentiation with previously published outputs (Trincado *et al.* 2018). In this dataset, SUPPA2 detected a smaller number of skipped exon events, 37,432, of which 481 were significant (*BH adjusted P* < .05), covering 374 unique genes. We then ran Splitpea on these results, leaving all other parameters at their defaults. The resulting rewired network involved 1,234 unique genes, with more potential gain-of-function edges than loss edges (836 gain, 629 loss, 18 mixed). Similar to the rMATS analysis, Splitpea identified additional genes that may be functionally affected by splicing changes, this time nearly a three-fold increase (1,103 genes) over those that SUPPA flagged as significantly spliced. While we do not repeat the downstream enrichment analysis for brevity, the same workflow can be applied to analyze the functional implications of the genes affected by gain or loss edges. To highlight the alternative network visualization capability of the package, we used the plotting function to export the network as a GML file and render the largest connected component with Cytoscape and the Prefuse Force-Directed layout (Fig. 1C).

## 4 Conclusion

Splitpea currently focuses on skipped exon events and infers splicing-driven PPI rewiring at the gene level, rather than constructing transcript- or isoform-specific interaction networks. This design choice enables Splitpea to operate directly on widely available short-read RNA-seq data without requiring full-length isoform resolution. As long-read sequencing and isoform-resolved splicing quantification become more broadly available, the same domain-based framework underlying Splitpea can be used to obtain higher fidelity results. Additional directions for future work include extending Splitpea to other splicing event classes (e.g., alternative splice site usage, intron retention) and incorporating transcript expression levels or uncertainty-aware splicing estimates to further refine interaction weighting and confidence.

In its current implementation, the Splitpea package serves as a valuable complement to existing differential splicing analysis pipelines, enabling users to directly investigate the functional impacts of skipped exon events. The generated rewired PPI networks can be easily analyzed and visualized with the integrated plotting and stats functions in the package. Additionally, the bundled reference files for human and mouse PPI networks, DDIs, and easily downloadable normal human tissue backgrounds allow users to quickly integrate Splitpea into workflows that already use rMATS or SUPPA2, facilitating a deeper interpretation of differential splicing changes that was not previously possible.

## Supplementary Material

btag154_Supplementary_Data

## Data Availability

The data and code underlying this article are available on GitHub (https://github.com/ylaboratory/splitpea-package) and can be accessed under the BSD 3-Clause License. The archived version of the package used to generate the results reported in this article is available on Zenodo  (https://zenodo.org/records/19194524).

## Supplementary material

Supplementary material pertaining to the example case studies described herein are made available at *Bioinformatics* online.
