# Supplementary material for: Splitpea: a Python package for protein-protein interaction network rewiring analysis due to alternative splicing
Source: Bioinformatics. 2026 Apr 8;42(4):btag154. doi: 10.1093/bioinformatics/btag154 (PMC13108719; doi:10.1093/bioinformatics/btag154)
Supplement: btag154_Supplementary_Data [file btag154_supplementary_data.zip › splitpea_package_supplement.pdf]

Supplementary Materials for *Splitpea: a Python package for protein-protein interaction network rewiring analysis due to alternative splicing*

Algorithm 1 Splitpea algorithm from Dannenfelser and Yao [1].

for each PPI between g_u, g_v **do**
    $\Psi^{(u)} :=$ significant exons for gene u
    $\Psi^{(v)} :=$ significant exons for gene v
    $D^{(u)} := \left\{ d_u \mid d_u \in g_u,\ \exists\, \text{exon}_i \text{ s.t. } \text{exon}_i \in \Psi^{(u)} \ \&\ \text{exon}_i \in d_u \right\}$
    $D^{(v)} := \left\{ d_v \mid d_v \in g_v,\ \exists\, \text{exon}_i \text{ s.t. } \text{exon}_i \in \Psi^{(v)} \ \&\ \text{exon}_i \in d_v \right\}$
    $w_{uv} :=$ network rewiring edge weight between g_u, g_v
    $\delta_{uv} :=$ direction classification of network rewiring between g_u, g_v
    for each DDI between $d_u \in D^{(u)}$ and $d_v \in D^{(v)}$ **do**
        $\Delta\psi_{d_u} := \min(\left\{ \Delta\psi_i \;\middle|\; \text{exon}_i \text{ s.t.exon}_i \in \Psi^{(u)} \ \&\ \text{exon}_i \in d_u \right\})$
        $\Delta\psi_{d_v} := \min(\left\{ \Delta\psi_i \;\middle|\; \text{exon}_i \text{ s.t.exon}_i \in \Psi^{(v)} \ \&\ \text{exon}_i \in d_v \right\})$
if $\forall d_u, d_v \in \text{DDI},\ \Delta\psi_{d_u} > 0 \ \&\ \Delta\psi_{d_v} > 0$ **then**
    $\delta_{uv} = \text{positive}$
else if $\forall d_u, d_v \in \text{DDI},\ \Delta\psi_{d_u} < 0 \ \&\ \Delta\psi_{d_v} < 0$ **then**
    $\delta_{uv} = \text{negative}$
else
    $\delta_{uv} = \text{chaos}$
    $w_{uv} = \frac{1}{|D^{(u)}|+|D^{(v)}|} \left(\sum_{d \in D^{(u)}} \Delta\Psi^{(u)} \ +\ \sum_{d \in D^{(v)}} \Delta\Psi^{(v)} \right)$
    return w_{uv}, δ_{uv}

Supplementary Tables

Supplementary Table 1: **Splitpea's runtime scales linearly.** Splitpea was run on the rMATS data from the PC3E-GS689 dataset [2] on Google Colab (AMD EPYC 7B12 CPU, 12 GiB RAM) using randomly subsampled event files (1%, 25%, 50%, 75%, and 100% of skipped exon events). Runtime is reported in seconds, and increases linearly with the number of differentially expressed exons ($R^2 = 0.99$).

**events used (%)**	**# skipped exons**	**runtime (s)**
1%	213	13.243
25%	5,420	78.171
50%	10,679	146.580
75%	15,538	214.800
100%	20,067	284.733

Supplementary Table 2: **External validation with known isoform-specific PPIs.** We compared Splitpea's rewired network built using rMATS data from the PC3E-GS689 dataset [2] against IntAct's curated cancer interaction set supported by experimental evidence [3]. After restricting IntAct to isoform-specific PPIs (730 unique PPIs), we observed a significant enrichment of 29 overlapping gene-level interactions with the Splitpea network (hypergeometric $p = 0.002$). Examples of rewiring-consistent isoform-specific interactions includes PAK1–PRKCZ (PRKCZ isoforms Q05513-1 and Q05513-2, with PAK1 Q13153) and FGFR1–FGF2 (e.g., P11362-14–P09038 and P11362–P09038-2). Uniprot IDs are provided for the isoforms corresponding to each gene.

Gene A	Gene B	Isoform A	Isoform B	Reference
TRIOBP	TRIOBP	Q9H2D6-5	Q9H2D6-5	Li et al. (2007) [4]
VHL	HIF1A	P40337-3	Q16665	Bex et al. (2007) [5]
ELOC	VHL	Q15369	P40337-3	Bex et al. (2007) [5]
VHL	EPAS1	P40337-3	Q99814	Bex et al. (2007) [5]
ID1	TCF3	P41134	P15923-1	Terai et al. (2000) [6]
ID2	TCF3	Q02363	P15923-1	Terai et al. (2000) [6]
TCF3	ID3	P15923-1	Q02535	Terai et al. (2000) [6]
TCF3	MYOD1	P15923-1	P15172	Terai et al. (2000) [6]
TCF3	TCF3	P15923-1	P15923-1	Terai et al. (2000) [6]
RAF1	KRAS	P04049	P01116-2	Alvarez-Moya et al. (2010) [7]
PPP2R5C	PPP2R1A	Q13362-1	P30153	Shouse et al. (2011) [8]
PPP2R5C	PPP2CA	Q13362-1	P67775	Shouse et al. (2011) [8]
GLI1	RPS6KB1	P08151	P23443-2	Wang et al. (2012) [9]
NRP1	VEGFA	O14786-2	P15692-4	Cecchi et al. (2012) [10]
HRAS	PIK3CD	P01112	O00329-2	Fransson et al. (2012) [11]
FGFR1	FGF2	P11362-14	P09038	Bono et al. (2013) [12]
MAPK14	MAPK1	Q16539-3	P28482	von et al. (2012) [13]
RAF1	MAP2K1	P04049-1	Q02750	Lito et al. (2014) [14]
SHC1	ERBB3	P29353-7	P21860	Petschnigg et al. (2014) [15]
COP 1	CUL4A	Q8NHY2-1	Q13619	Lu et al. (2014) [16]
COP 1	DDB1	Q8NHY2-1	Q16531	Lu et al. (2014) [16]
NFKBIA	RELA	P25963	Q04206-1	Liu et al. (2015) [17]
PAK1	PRKCZ	Q13153	Q05513-1	Jinesh et al. (2016) [18]
PAK1	PRKCZ	Q13153	Q05513-2	Jinesh et al. (2016) [18]
FGFR1	FGF2	P11362	P09038-2	Ronca et al. (2015) [19]
EFNB2	EPHB4	P52799	P54760-1	Pradeep et al. (2015) [20]
ERBB2	PTPN18	P04626	Q99952-1	Wang et al. (2014) [21]
NRG1	EGF	Q02297-6	P01133	Freed et al. (2017) [22]
BCL2L1	BAX	Q07817-1	Q07812	Chu et al. (2018) [23]

Supplementary Figures

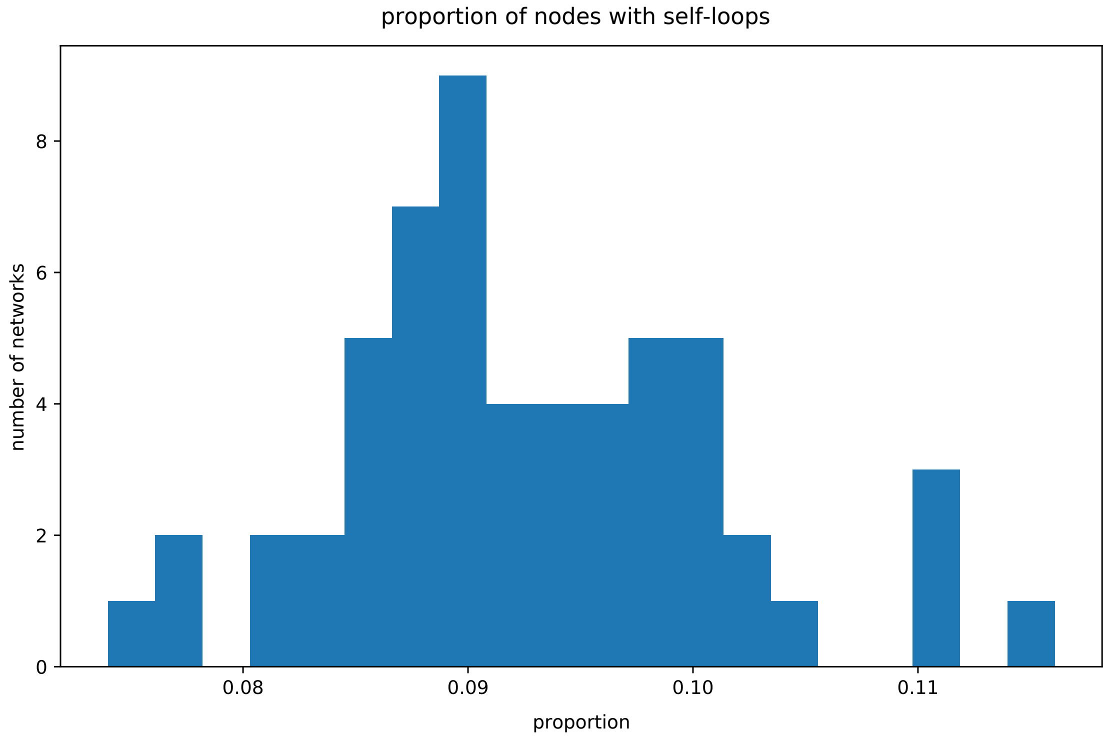

Supplementary Figure 1: **Self-loop prevalence across UCS vs uterus networks.** Percentage of nodes with a self-loop in each of the 57 gene-level Splitpea networks from the UCS vs uterus comparison used in the GitHub tutorial; self-loops are infrequent overall (mean 9.3% of nodes per network)

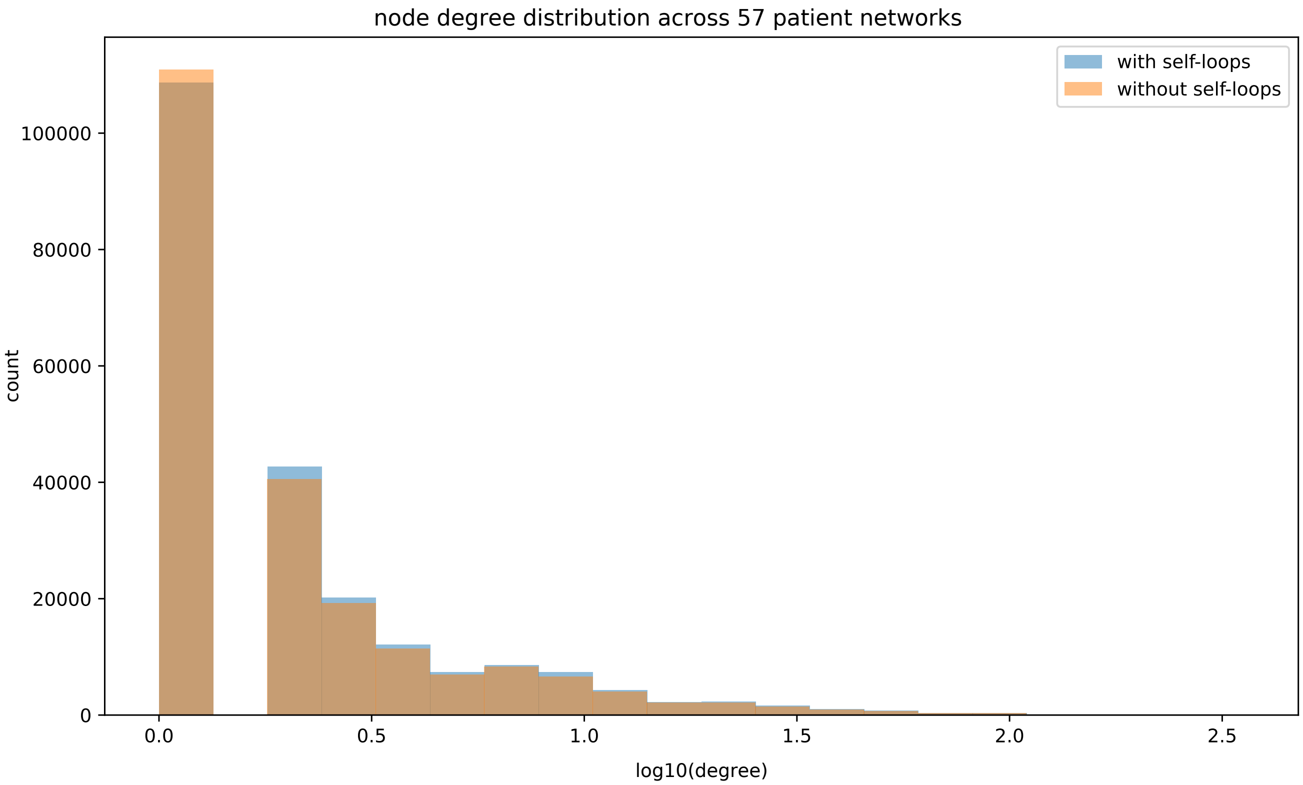

Supplementary Figure 2: **Degree distributions with versus without self-loops.** Node degree distributions computed across the 57 Splitpea networks from the UCS vs uterus comparison used in the GitHub tutorials including self-loops versus after removing self-loops; the distributions largely overlap, and any differences reflect at most a one-degree shift.

References

1. Dannenfelser, R. & Yao, V. Splitpea: Quantifying Protein Interaction Network Rewiring Changes Due to Alternative Splicing in Cancer. *Pacific Symposium on Biocomputing. Pacific Symposium on Biocomputing* **29,** 579–593. ISSN: 2335-6936 (2024).
2. Wang, Y. *et al.* rMATS-turbo: An Efficient and Flexible Computational Tool for Alternative Splicing Analysis of Large-Scale RNA-seq Data. *Nature Protocols* **19,** 1083–1104. ISSN: 1750-2799 (Apr. 2024).
3. Orchard, S. *et al.* The MIntAct project—IntAct as a common curation platform for 11 molecular interaction databases. *Nucleic acids research* **42,** D358–D363 (2014).
4. Li, X. *et al.* Expression, purification, and characterization of Tara, a novel telomere repeat-binding factor 1 (TRF1)-binding protein. *Protein expression and purification* **55,** 84–92 (2007).
5. Bex, C., Knauth, K., Dambacher, S. & Buchberger, A. A yeast two-hybrid system reconstituting substrate recognition of the von Hippel-Lindau tumor suppressor protein. *Nucleic acids research* **35,** e142–e142 (2007).
6. Terai, S., Aoki, H., Ashida, K. & Thorgeirsson, S. S. Human homologue of maid: A dominant inhibitory helix-loop-helix protein associated with liver-specific gene expression. *Hepatology* **32,** 357–366 (2000).
7. Alvarez-Moya, B., Lopez-Alcala, C., Drosten, M., Bachs, O. & Agell, N. K-Ras4B phosphorylation at Ser181 is inhibited by calmodulin and modulates K-Ras activity and function. *Oncogene* **29,** 5911–5922 (2010).
8. Shouse, G., Nobumori, Y., Panowicz, M. & Liu, X. ATM-mediated phosphorylation activates the tumor-suppressive function of B56γ–PP2A. *Oncogene* **30,** 3755–3765 (2011).
9. Wang, Y. *et al.* The crosstalk of mTOR/S6K1 and Hedgehog pathways. *Cancer cell* **21,** 374–387 (2012).
10. Cecchi, F. *et al.* Targeted disruption of heparan sulfate interaction with hepatocyte and vascular endothelial growth factors blocks normal and oncogenic signaling. *Cancer cell* **22,** 250–262 (2012).
11. Fransson, S. *et al.* p37δ is a new isoform of PI3K p110δ that increases cell proliferation and is overexpressed in tumors. *Oncogene* **31,** 3277–3286 (2012).
12. Bono, F. *et al.* Inhibition of tumor angiogenesis and growth by a small-molecule multi-FGF receptor blocker with allosteric properties. *Cancer cell* **23,** 477–488 (2013).
13. Von Brandenstein, M., Schlosser, M., Richter, C., Depping, R. & Fries, J. ETS-dependent p16INK4a and p21waf1/cip1 gene expression upon endothelin-1 stimulation in malignant versus and non-malignant proximal tubule cells. *Life Sciences* **91,** 562–571 (2012).
14. Lito, P. *et al.* Disruption of CRAF-mediated MEK activation is required for effective MEK inhibition in KRAS mutant tumors. *Cancer cell* **25,** 697–710 (2014).
15. Petschnigg, J. *et al.* The mammalian-membrane two-hybrid assay (MaMTH) for probing membrane-protein interactions in human cells. *Nature methods* **11,** 585–592 (2014).
16. Lu, G. *et al.* Phosphorylation of ETS1 by Src family kinases prevents its recognition by the COP1 tumor suppressor. *Cancer cell* **26,** 222–234 (2014).
17. Liu, B. *et al.* A cytoplasmic NF-κB interacting long noncoding RNA blocks IκB phosphorylation and suppresses breast cancer metastasis. *Cancer cell* **27,** 370–381 (2015).

18. Jinesh, G. *et al.* Blebbishields, the emergency program for cancer stem cells: sphere formation and tumorigenesis after apoptosis. *Cell Death & Differentiation* **20,** 382–395 (2013).

19. Ronca, R. *et al.* Long-pentraxin 3 derivative as a small-molecule FGF trap for cancer therapy. *Cancer cell* **28,** 225–239 (2015).

20. Pradeep, S. *et al.* Erythropoietin stimulates tumor growth via EphB4. *Cancer cell* **28,** 610–622 (2015).

21. Wang, H.-M. *et al.* The catalytic region and PEST domain of PTPN18 distinctly regulate the HER2 phosphorylation and ubiquitination barcodes. *Cell research* **24,** 1067–1090 (2014).

22. Freed, D. M. *et al.* EGFR ligands differentially stabilize receptor dimers to specify signaling kinetics. *Cell* **171,** 683–695 (2017).

23. Chu, C.-W. *et al.* GSK3β-mediated Ser156 phosphorylation modulates a BH3-like domain in BCL2L12 during TMZ-induced apoptosis and autophagy in glioma cells. *International journal of molecular medicine* **42,** 905–918 (2018).
